# The impact of an exercise intervention using low-cost equipment on functional fitness in the community-dwelling older adults: A pilot study

**DOI:** 10.3389/fphys.2022.1039131

**Published:** 2022-10-17

**Authors:** Filipe Rodrigues, Nuno Amaro, Rui Matos, Diogo Mendes, Diogo Monteiro, Pedro Morouço

**Affiliations:** ^1^ ESECS, Polytechnic of Leiria, Leiria, Portugal; ^2^ Life Quality Research Centre (CIEQV), Leiria, Portugal; ^3^ Research Center in Sport, Health and Human Development (CIDESD), Vila Real, Portugal; ^4^ Polytechnic of Leiria, ciTechCare—Center for Innovative Care and Health Technology, Leiria, Portugal

**Keywords:** fitness, experimental, autonomy, frailty, older adults

## Abstract

Exercise interventions have shown significant improvements in the older adult population regarding functional and cognitive capacity. However, much research has applied exercise protocols that are expensive both for research and participation. Thus, high investments that are made are currently not ecologically efficient. The aim of the study was to determine whether the impact of a 20-week multicomponent exercise intervention using low-cost material could improve physical fitness in community-dwelling older adults. We tested the feasibility of this three times per week exercise protocol using an inclusive approach. Eligibility criteria included age ≥65 years, the ability to stand and walk with or without assistive devices, being physically inactive; medical clearance, and living in the community. Outcomes were muscle resistance measured using the chair-stand test for lower limbs, cardiorespiratory capacity measured using the 6-min walk test, and agility and balance using the Timed-up and Go test. Flexibility was measured using the chair sit and reach for lower limbs and the back scratch for upper limbs using the dominant leg and arm, respectively. Hand grip strength was measured using a dynamometer. Thirty participants (*n* = 30) were recruited and included in the intention-to-treat analysis. The mean age of participants was 70 years (±3.62 years), 100% were Caucasians, and 50% were female. There was a significant trend toward a clinically important improvement in the chair-stand (t = −5.23; *p* < 0.001; d = 0.36), arm curl (t = −5.21; *p* < 0.001; d = 0.74), 6-min walk test (t = −4.69; *p* = <0.001; d = 0.77), timed-up and go test (t = 8.788; *p* < 0.001; d = 1.18), and hand grip strength (t = 2.846; *p* = 0.009; d = 0.23). There were also differences in the back scratch (t = 2.243; *p* = 0.035; d = 0.29) and chair sit and reach test (t = −3.380; *p* < 0.001; d = 0.15). This pilot study has provided preliminary evidence that a 20-week community-based low-cost exercise program may be effective in improving overall functional fitness in older people. The pilot trial has provided the necessary data to design future randomized-controlled trials that can be implemented in the community in an ecological feasible manner.

## Introduction

The number of older adults is increasing worldwide and most wish to live in their homes rather than living in nursing homes or daycare centers (Sun et al., 2021). To achieve this, the elderly will require community programs and policies that provide exercise interventions to remain living independently with acceptable physical and functional fitness. These programs are often provided only for research purposes with prior-selected inclusion criteria and end when data collection is finished. This decision while acceptable for studying the effect of exercise in specific populations limits the inclusiveness of exercise that has been marginalized worldwide. In other instances, exercise programs are structured considering high-cost resistance and cardiorespiratory machines (i.e., treadmills) which is not ecologically feasible as funds are limited. Community Exercise programs supervised and supported by exercise physiologists for older adults, using low-cost equipment like those used in their daily activities could be an opportunity to promote functional fitness. Thus, the search for reliable long-term and low-cost exercise interventions is warranted aiming to increase physical and functional fitness in the elderly.

Exercise applied in early aging adults can prevent functional decline and is associated with increased autonomy and independence, and a lower burden on health care (Paterson and Warburton, 2010). Its effects are not limited to functional capacity but also have positive implications for reducing fall risk and fall-related injuries (Wang et al., 2020), reducing clinical depression (Miller et al., 2020), and increasing sleep quality (Fank et al., 2022) in older adults.

The specific benefits of an acute multicomponent exercise intervention consisting of muscular resistance, cardiorespiratory, balance, and agility exercises to attenuate the functional decline in older adults have been described in the literature (American College of Sports Medicine et al., 2009; Izquierdo et al., 2021; Mende et al., 2022). This training mode has been described as a significant contributor in improving functional fitness, metabolic-related outcomes, and cognitive performance (Monteiro et al., 2022). Thus, by improving significantly physiological and psychological outcomes, it is expected that the quality of life may be improved also (Oliveira et al., 2021) expecting to increase or maintain independent living and autonomy (Campbell et al., 2021; Rodrigues et al., 2022). However, most reported interventions consist of including resistance training using expensive equipment that requires funds that are not always available for community purposes (Fragala et al., 2019; Rodrigues et al., 2022).

To the best of our knowledge, this type of intervention has been insufficiently implemented in the community in individuals of advanced age (García-Molina et al., 2018; Oh et al., 2021). More research is required to fully understand the extent to which exercise using low-cost equipment improves or at least retains functional fitness. Since exercise provides long-term quality of life (Campbell et al., 2021), such an approach may help stakeholders to offer efficient interventions to delay or even attenuate age-related functional decline, promoting health-related quality of life in community-living older adults. In addition, community exercise programs with an inclusive approach could attract more older adults to participate in regular exercise. The application of these exercise programs should be long-term oriented for empirical evidence and not limited to research.

The aim of the study was to determine whether the impact of a multicomponent exercise intervention using low-cost material could improve physical fitness in community-dwelling older adults. We evaluated participants functional fitness and associated measures of quality of life using low-cost valid and reliable measures. It was hypothesized that the exercise intervention could have a positive and significant effect on physical fitness in the community-dwelling older adults.

## Materials and methods

### Design

The study was a 20-week quasi-experimental trial performed according to the SPIRIT 2013 and the CONSORT statement for transparent reporting. The study was conducted from 1 October 2021, to 28 February 2022, in a 20 m × 15 m open space of the School of Education and Social Sciences (Polytechnic Institute of Leiria, Leiria, Portugal). The study followed the principles of the Declaration of Helsinki (World Medical Association, 2013) and was approved by the Polytechnic Institute of Leiria and the Life Quality Research Center (reference number: UIDB/04748/2020). All participants provided written informed consent. There was no financial compensation.

### Participants

The G*Power 3.1 (Faul et al., 2009) was used to calculate the required sample size, including the following parameters: effect size = 0.7, *α* = 0.05, and statistical power = 0.95, suggesting a minimum of 24 participants, which was respected in this study.

A particular challenge of elderly research is recruiting the study population, considering the current means of communication. Hence, we used social media platforms, flyers, and regional journals to publicize the exercise intervention. Our approach was to recruit as many potential participants living in the community as possible, using an inclusive approach. All participants were included in the exercise program group. Nonetheless, for objective and safety purposes, some inclusion criteria had to be followed. Inclusion criteria were age ≥65 years, the ability to stand and walk with or without assistive devices, being physically inactive; and living in the community. Assessments were carried out by a certified exercise physiologist with the assistance of a senior researcher at baseline and after the intervention. Intra-observer validity was ensured using Cohen’s kappa. The inter-rater agreement presented good agreement (k = 0.78). All co-existing diseases or conditions related to the intervention will be treated in accordance with prevailing medical practice and will be reported as adverse events.

### Intervention

The exercise protocol followed the FITT principles (American College of Sports Medicine, 2021). The exercise intervention was programmed in 3-morning sessions per week, light-to-moderate intensity-oriented exercises, during 45–60 min of resistance, cardiorespiratory, balance, agility, and flexibility training. An experienced exercise physiologist with in-depth training on older adults’ exercise prescription supervised all sessions and provided supportive instructions and encouragement.

Exercise sessions were done in a day-off-day sequence, on weekdays in the morning according to the preference of the participants. Two groups were created for safety measures due to the number of participants. The intensity was measured using the talk test, which is a valid, reliable, practical, and inexpensive tool for prescribing and monitoring exercise intensity (Reed and Pipe, 2014). During training sessions, the exercise physiologist applied the talk test to each participant immediately after each component and at the end of the session. The time of each session varied between 45 and 60 min according to the supervised training protocol. Three different sessions were created and applied to provide a different stimulus to the participants. Resistance exercises were performed using bodyweight, ankle weights, and dumbbell equipment. Chair squats, leg extension, standing leg curl, hip flexion, and standing or seated calf raise for lower body muscles were performed. Lateral raise, bicep curl, front raise, shoulder press, seated row, and shoulder shrug for upper body muscles were performed. Exercises were performed in a circuit and performed at a 1:1 ratio. Participants completed as many repetitions as possible for 30–60 s and rested equal time. The number of circuits increased for maintaining stimulus every 4 weeks. In total, resistance training lasted between 20 and 30 min. Regarding cardiorespiratory fitness, different activities were developed. Dual-task exercises were conducted such as: walking while counting a three-digit number forward; walking while counting from 100 backward; walking while memorizing three to five words. Duration for this component ranged from 10 to 15 min according to the exercise program. Throwing and/or catching as well as single-leg static and dynamic exercises using softballs were performed for training balance and agility. Safety measures were considered and thus distance between each participant was defined and the duration for each activity ranged from 5 to 10 min. Dynamic stretching exercises were performed at the end of the training session. Flexibility exercises focused low intensity stretching on recruited muscles during previously executed muscles and were performed at a 1:1 ratio. Duration for this component ranged from 5 to 10 min according to the exercise program.

The exercise intervention was designed to meet the exercise and physical activity guidelines for older adults established by the American College of Sports Medicine and the American Heart Association (Nelson et al., 2007). Training attendance was recorded every session. Adherence to the exercise intervention program was documented in a weekly register by the exercise physiologist. We considered persistent participants those who attended at least 70% of the total training sessions.

The costs drawn from the intervention were basically those generated by hiring one exercise physiologist for the intervention and the collaboration of a researcher who shared their work 3 days a week for the duration of the study. An initial investment of €1055 (USD $1040.20; currency dated on September 22nd) was made to buy the equipment. The total amount of investment was used to buy twenty sets with different resistance and weights of dumbbells, ankle weights, elastic bands, kettlebells, and softballs. Details of the investment of each material is reported in the supplemental material (Supplementary Table S1).

### Outcome measures

The outcomes were muscle resistance measured using the chair-stand test for lower limbs and the arm curl using the dominant arm for upper limbs. Cardiorespiratory capacity was measured using the 6-min walk test, and agility and balance using the Timed-up and Go test. Flexibility was measured using the chair sit and reach for lower limbs and the back scratch for upper limbs using the dominant leg and arm, respectively. These tests demonstrate good agreement with measures of functional ability (Rikli and Jones, 2013) and are considered valid and reliable measures of functional fitness in the elderly. Hand grip strength was measured using a dynamometer (CAMRY EH101, Sensun Weighing Apparatus Group Ltd., Guangdong, China) which has been validated for this population (Huang et al., 2022). We also recorded the possible number of falls, visits to the emergency, hospitalization, and length of stay.

### Statistical analysis

Normality of distribution was checked using the Shapiro-Wilk at baseline. Means (M) and Standard-Deviations (SD) are reported. Percentages of ethnicity and gender, as well as protocol attendance are also reported. The paired sample *t*-test to examine differences at baseline and after the intervention was performed. Cohen’s d effect size was calculated between time points and thresholds were set at 0.2, 0.5, and 0.8 for small, medium, or large effects, respectively. Significance was set at *p* < 0.05, and all analyses were performed under the intention-to-treat principle. We analyzed data using IBM SPSS Statistics for Windows, Version 25.0 (IBM Corp., Armonk, NY).

## Results

Thirty participants were included in the exercise intervention. The mean age of participants was 70 years (±3.62 years), 100% were Caucasians, and 50% were female. While there were no dropouts recorded from the exercise program, missing completely at random values were identified. We used the expectation-maximization approach to handle missing data. * Mean attendance rates were 75%, ranging from 70% to 100%. Data at baseline displayed normal distribution (Table 1). No falls were reported during the exercise program. There was a significant trend toward a clinically important improvement in the chair-stand, arm curl, 6-min walk test, timed-up and go test, and hand grip strength (*p* < 0.01) with small and large effect sizes ranging from d = 0.15 to d = 1.18, respectively. There were also differences in the back scratch and chair sit and reach test (*p* < 0.05). Medium effect sizes for flexibility tests were 0.23 for chair sit and reach and 0.29 for back scratch. The 6-min walk test displayed the most change between baseline and after intervention (Table 1).

**TABLE 1:** Mean differences between time points in all outcomes.

	Mean (SD)	S-W	Mean (SD)	Change	*t*	*p*	*d*
	Baseline		Follow-up				
Chair-stand test (repetitions)	14.86 (4.39)	0.145	16.42 (4.26)	1.56 (0.13)	−5.230	<0.001	0.36
Arm curl (repetitions)	22.85 (5.04)	0.521	27.65 (6.70)	4.80 (1.66)	−5.217	<0.001	0.74
6-min walk test (m)	579.63 (42.03)	0.550	616.73 (53.48)	37.10 (11.45)	−4.697	<0.001	0.77
Timed-up and go (s)	5.30 (0.64)	0.325	4.64 (0.46)	−0.66 (0.18)	8.788	<0.001	1.18
Chair sit and reach (cm)	0.15 (12.72)	0.190	−2.87 (13.43)	−3.02 (0.71)	2.846	0.009	0.23
Back scratch (cm)	4.07 (12.62)	0.376	0.46 (11.47)	3.61 (1.15)	2.243	0.035	0.29
Hand grip strength (kg)	31.57 (8.11)	0.129	32.82 (8.47)	1.25 (0.36)	−3.380	<0.001	0.15

Notes: S-W, Shapiro Wilk; *t*, paired sample *t*-test; d, Cohen’s d.

*We compared the results from the paired-wise *t*-test between raw and imputed sets and the results were not significantly different. Thus, we decided to report the results from the raw data set for transparency.

Related to number of falls and fall-related injuries, the exercise physiologists did not report any falls during exercise intervention. Participants also reported zero falls outside the exercise program. No emergencies and hospitalizations were reported.

## Discussion

The aim of the study was to determine whether the impact of a multicomponent exercise intervention using low-cost equipment could improve physical fitness in community-dwelling older adults. Our results showed that a multicomponent exercise intervention using low-cost equipment including low-to-moderate intensity resistance, cardiorespiratory, balance, agility, and flexibility training exercises performed for 20 weeks, three times per week, provides a significant benefit and can help to reverse the functional decline associated with aging. Thus, our results confirm the proposed hypothesis of a positive and significant of the exercise intervention on physical fitness in the community-dwelling elderly.

With a very small investment in equipment, subjects were still able to achieve significant improvements in different functional capacities compared to traditional strength and/or cardiorespiratory training using high-cost machines. Indeed, improvements were quite like those expressed in a recent review with 28 studies focused on resistance training (Lopez et al., 2021). While a complete improvement is favorable, expressed gains were more noticed in the time-up-and-go test. With a 20-week program of concurrent training with and without repetitions to failure, 37 males were able to increase their muscle strength, but not their agility (Teodoro et al., 2019). Accordingly, it may be relevant to develop multi-component programs, as there are undeniable associations between high physical activity and low sedentary behavior with better muscle strength and power in physically active older adults (Ramsey et al., 2021). As described in a recent meta-analyses (Borges-Machado et al., 2021; Li et al., 2022) multicomponent exercise programs are efficient in producing improvement in several aspects of health-related physical fitness.

Existing literature has showed that regular exercise using resistance machines and cardio machines can increase cardiorespiratory capacity, muscle strength and muscular endurance in older individuals (ACSM et al., 2009; ACSM, 2021), and results from experimental studies testing this assumption have confirmed (García-Molina et al., 2018; Monteiro et al., 2022; Oh et al., 2021). We support these claims and the results from our study are similar related to the effects of exercise on physical fitness compared to previous studies. In some health-related physical fitness components such as muscular resistance and cardiorespiratory capacity, our exercise program seems to have greater effect compared to other studies using high-cost equipment exercise protocols (Monteiro et al., 2022; Teodoro et al., 2019). Our study also corroborates with the results from meta-analytic studies (Borges-Machado et al., 2021; Frank et al., 2022) considering exercise interventions using high-cost equipment on physical fitness components, showing that exercise can increase health-related indicators in community-dwelling older adults. Thus, the question of what equipment is best suited to the needs and abilities of older adults is warranted. While resistance machines, body weight, free weights (i.e., dumbbells and adjustable ankle weights), exercise bands, or a combination of all of the above is desirable, the access and availability is not always possible. Thus, while the applicability of our program showed equal or better results related to physical fitness compared to previous studies using machines (e.g., Oliveira et al., 2021) further testing is paramount as the comparison of exercise intensity and active time vary according to active muscle contraction between bodyweight and resistance machine exercise.

With accumulating evidence emphasizing exercise as a strong predictor of positive health outcomes in older adults, studies have sought to design and test multicomponent interventions to improve functional fitness and decrease falling risk and fall-related injuries (García-Molina et al., 2018; Oh et al., 2021). To date, research has shown the short-term benefits of exercise interventions, whereas the long-term effects of these protocols are not well established (Puts et al., 2017). In this study we were able to improve physical fitness, by combining a multicomponent exercise program with low-cost equipment, and maintain autonomy, as there were no falls associated with this intervention, nor did participants report falls on other occasions. There are several potential explanations for the success of our intervention. We provided a community-based exercise program with an inclusive approach for older adults, who might have greater potential to participate and who intend to still live in their homes as reported in other studies (Campbell et al., 2021; Oh et al., 2021). Moreover, large group dynamics from exercise sessions may have caused positive feedback, as well as reinforcement from the celebration of high attendance, which may have maximized adherence to the exercise program.

Current results can provide meaningful design and implementation of a public health intervention for older adults living in the community. Implementing an exercise program in the community could decrease potential health care costs related to aging (e.g., falls, hospitalization, medications). Long-term systematic interventions should examine the efficacy of these programs, especially across communities with different characteristics. Not only the exercise interventions need to be inclusive, but the implementation of these programs should also be oriented towards the needs of this population (Monteiro et al., 2022). Considering the need for funds to implement an exercise program, the search for reducing cost but maintaining efficiency with significant adherence is needed. People not only want to leave more years, but they also want to live with independence and quality.

## Limitations

Our study has some limitations. Due to the sample size and objective, this was a quasi-experimental study. Thus, the results from this study should be further explored, since there is no certainty that improving functional fitness will translate into better long-term outcomes using a randomized controlled approach. In addition, we did not compare the effect of exercise interventions using low-cost vs. high-cost equipment. More studies are warranted to examine the efficacy of this type of intervention using low-cost materials. While we did not have a control group to compare the effects of the exercise program on physical fitness, in pilot studies such as this in which we provide initial evidence of an low-cost exercise program. Our study, nevertheless, has several strengths, including its novelty. Most exercise interventions in older adults have been performed in laboratory settings, diminishing their replicability in the community. Future research should continue to work on educational programs that move from experimental settings to a real-world context.

## Conclusion

A low-cost multicomponent exercise program proved to be safe and effective in improving functional fitness associated with aging in community-dwelling elderly. It was also shown to be of interest for the elderly since adherence was above 70% regarding the 20-week protocol. These findings open the possibility for a shift from the traditional resistance and cardiorespiratory machines approach in laboratory or gym settings for elders to one that is inclusive and community-based and that can be cost-effective and of simple implementation for all older adult populations.

## Data Availability

The raw data supporting the conclusions of this article will be made available by the authors, without undue reservation.
