# Supplementary material for: The impact of an exercise intervention using low-cost equipment on functional fitness in the community-dwelling older adults: A pilot study
Source: Front Physiol. 2022 Oct 17;13:1039131. doi: 10.3389/fphys.2022.1039131 (PMC9621329; doi:10.3389/fphys.2022.1039131)
Supplement: Supplementary file 1 [file Table1.pdf]

# *Supplementary Material*

## 1 Table S1. Details of the investment of each material

| Equipment | Unit | Price/Unit Euro (€) | Price/Unit USD ($) | Cost Euro (€) | Cost USD ($) |
|---|---|---|---|---|---|
| Dumbbell 1.00 kg | 20 | 2.95 | 2,92 | 59,00 | 58,40 |
| Dumbbell 2.00 kg | 20 | 5.58 | 5,52 | 111,60 | 110,40 |
| Ankle Weights 0.75 kg | 20 | 5.93 | 5,87 | 118,60 | 117,40 |
| Ankle Weights 1.25 kg | 20 | 7.55 | 7,47 | 151,00 | 149,40 |
| Ankle Weights 2.00 kg | 20 | 9.24 | 9,15 | 184,80 | 183,00 |
| Kettlebell 4.00 kg | 20 | 12.74 | 12,61 | 254,80 | 252,20 |
| Softball 12.50 cm | 20 | 1.66 | 1,64 | 33,20 | 32,80 |
| Softball 17.50 cm | 20 | 2.91 | 2,88 | 58,20 | 57,60 |
| Softball 20.00 cm | 20 | 4.19 | 4,15 | 83,80 | 83,00 |
| **TOTAL** | **180** | **-** | **-** | **1055.00 €** | **1044.20 $** |

***Notes:*** 1kg = 2.20lbs; 1cm = 39 inches
